# Influence of Endogenous and Exogenous Estrogenic Endocrine on Intestinal Microbiota in Zebrafish

**DOI:** 10.1371/journal.pone.0163895

**Published:** 2016-10-04

**Authors:** Yukun Liu, Yayun Yao, Huan Li, Fang Qiao, Junlin Wu, Zhen-yu Du, Meiling Zhang

**Affiliations:** 1 Laboratory of Aquaculture Nutrition and Environmental Health, School of Life Sciences, East China Normal University, Shanghai, 200241, China; 2 Nextomics Biosciences Co. Ltd., Wuhan, 430073, China; Chinese Academy of Sciences, CHINA

## Abstract

Gender is one of the factors influencing the intestinal microbial composition in mammals, but whether fish also have gender-specific intestinal microbial patterns remains unknown. In this decade, endocrine disrupting chemicals in surface and ground water of many areas and increasing observation of freshwater male fish displaying female sexual characteristics have been reported. Here we identified the difference in intestinal microbiota between male and female zebrafish, and revealed the influence of endocrine disrupting chemicals on zebrafish intestinal microbiota by using high-throughput sequencing. The results indicated that Fusobacteria, Bacteroidetes and Proteobacteria were dominant in the gut of zebrafish and there were no obvious gender-specific intestinal microbial patterns. Two endocrine disrupting chemicals, Estradiol (E2) and Bisphenol A (BPA), were selected to treat male zebrafish for 5 weeks. E2 and BPA increased vitellogenin expression in the liver of male zebrafish and altered the intestinal microbial composition with the abundance of the phylum CKC4 increased significantly. Our results suggested that because of the developmental character and living environment, gender did not influence the assembly of intestinal microbiota in zebrafish as it does in mammals, but exposure extra to endocrine disrupting chemicals disturbed the intestinal microbial composition, which may be related to changes in host physiological metabolism.

## Introduction

For most terrestrial mammals, gut microbiota are involved in absorbing nutrition [1], defending against etiological microbes [2] and even modulating social behaviors [3]. Bacterial dysbiosis has been shown to be closely related to the risk of gastrointestinal disease [4], genitourinary inflammation [5], pre-malignant lesions in the colon [6] and breast cancers [7]. In 1984, Adlercreutz *et al*. found that antibiotics could reduce the estrogen level and assumed that intestinal microbiota may relate to estrogen metabolism [8]. Since then, more and more evidences have shown that microbiota of the female and male animals respond to the same stimulation in distinct ways. For example, gender bias has been observed in numerous diseases such as inflammatory bowel disease and Type 1 diabetes (T1D) [9,10]. Removal of the microbiota reduced T1D incidence and increased the testosterone level in female mice [10], suggesting a complex but underlying interaction between microbiota and sex hormone level. However, the relationships among the intestinal microbiota, sex hormone and host health status have not been extensively investigated in animals other than terrestrial mammals [11].

In this decade, endocrine disrupting chemicals (EDCs) have been reported in surface and ground waters in many areas including Europe, Asia, South America and Oceania[12]. Freshwater male fish display female sexual characteristics due to the EDC pollution. The UK Government’s Environment Agency found in 2004 that 86% of male fish sampled at 51 sites around the country were intersex [13]. Considering the close relationship between hormone and gut microbiota in terrestrial mammals and the increasing observation of sexual disruption in fish [14], we wondered whether the “microgenderome”, i.e. sex hormone modulation associated with the microbiome [9], exists in fish and whether EDCs could modify the microbiota.

Estradiol (E2), as a natural EDC, functions via estrogen receptors (ERα and ERβ). It increases vitellogenin (VTG) level, which results in transsexual male fish and genital deformities [15]. Bisphenol A (BPA), a common synthetic EDC, is found in a multitude of products including food, beverage packaging, flame retardants, adhesives, building materials, electronic components and paper coatings [16]. BPA, a nonsteroidal xenoestrogen, exhibits approximately 1/10^4^ the activity of estradiol [17]. The effects of E2 and BPA on the microbiota of fish remain unknown.

In this study, zebrafish (*Danio rerio*) was selected as the model. Differences in intestinal microbiota between male and female fish were characterized. In addition, the influences of E2 and BPA on the intestinal microbial composition were identified.

## Methods and Materials

### Experimental animals, design and facilities

Zebrafish were purchased from Yuyi tropical fish farm (Shanghai, China). The average weight was 0.40±0.024g. BPA was purchased from Sangon Biotech (Shanghai, China). E2 was purchased from Fluka (Mo, USA). Acetone was purchased from Lingfeng Chemical Reagent Co. Ltd. (Shanghai, China) and acetone was the vehicle for E2 and BPA. Serial concentrations of E2 (500ng/L and 2000ng/L) and BPA (200μg/L and 2000μg/L) were used for pre-trial[18,19]. Because of the pre-trial data (S1 Fig) and the short exposing time (5 weeks in the present study) compared with the long exposing time in natural water, BPA at 2000μg/L and E2 at 2000ng/L were selected for subsequent experiments.

The zebrafish were divided into four groups according to the treatment: Group F, female zebrafish were reared with 3.33mL/L acetone; Group M, male fish were reared with 3.33mL/L acetone; Group E2, male zebrafish were reared with 2000ng/L E2; Group BPA, male zebrafish were reared with 2000μg/L BPA. Only the male fish were treated with EDCs to avoid the effect of the female own estrogen. Group F and Group M occupied five tanks which contained 30 fish in each tank while Group BPA and Group E2 occupied four tanks which contained 30 fish in each tank. All the fish were reared for 5 weeks. During the whole experiment, one-third of the tank water was exchanged once per day with aerated water supplied BPA, E2, or acetone, as appropriate. The water quality parameters across the whole experiment were pH 7.5–7.9, temperature 26–28°C, dissolved oxygen 4.8–6.4 mg/L, and total ammonia nitrogen <0.02 mg/L. Zebrafish were fed three times per day, at 08:00, 16:00 and 22:00 h. Based on the amount of feed remaining on the following day, daily rations were adjusted to a feeding level slightly over satiation. Uneaten feed was removed daily with a siphon tube. The initial body length and weight of fish were similar and no significant difference was found at the end of the experiment (S2 Fig). All samples were collected at the end of the feeding trial following a 24 h starvation period. Etomidate was injected 0.3μg/g for anesthesia before the decapitation.

All experiments were conducted under the Guidance of the “Care and Use of Laboratory Animals in China”. This research was approved by the Committee on the Ethics of Animal Experiments of East China Normal University.

### VTG gene expression level in male zebrafish liver

The liver of zebrafish in Groups M, E2 and BPA were carefully divided from each fish. Five fish from each tank were collected as one sample in each group. Trizol reagent (Invitrogen, Carlsbad, CA, USA) was used to extract total RNA from liver samples. A Nano Drop 2000 (Thermo Scientific, Waltham, MA, USA) was used to measure the amount of RNA. Visualization of the 28S/18S ribosomal RNA ratio on a 1% agarose gel was used to assess the RNA quality of each sample. Total RNA (1μg) was used to synthesize complementary DNA using the PrimeScript™ RT Reagent Kit (Takara, Dalian, China), according to the manufacturer’s instructions. The forward and reverse primers for VTG gene expression quantification were, VTGF: 5ʹ-TTTTTGCTATCATCGCCCGT-3ʹ and VTGR: 5ʹ-TTCACCCAGGACACCAGCTT-3ʹ. The PCR program was run on a CFX Connect Real Time System (Bio-Rad, USA) with Ultra SYBR Mixture (CWBio, Beijing, China). The PCR program began with a 3 min denaturation step at 94°C; followed by 30 cycles of 1 min at 94°C (denaturation), a 1 min annealing and elongation collective step at 60°C. Housekeeping gene was β-actin, and the primers were ACTF: 5ʹ-TCTGGTGATGGTGTGACCCA-3ʹ and ACTR: 5ʹ-GGTGAAGCTGTAGCCACGCT-3ʹ. The quantification of gene expression was calculated by the comparative ΔΔCT method, VTG expression in each group was normalized to the endogenous reference β-actin level and reported as the fold difference relative to β-actin gene expression. All the assays were performed in triplicate and repeated at least twice. Data were expressed as mean ±standard error of the mean (SEM).

### Triglyceride content of male zebrafish muscle

TG content, which was one of the most important parameters reflecting the host lipid metabolism, was detected in this study. Because of the limited available quantity of zebrafish livers, triglyceride (TG) content was determined in muscles of fish from Groups M, E2 and BPA. About 0.034±0.010 g muscle was prepared for TG content test. A TG test kit (BHKT Clinical Reagent Co. Ltd., Beijing, China) was used. In brief, 20mL ethanol was added to each gram of tissue homogenate. Then the mixture was centrifuged at 2,000g for 10min at 4°C. The GPO-AAP method was performed to test the supernatant [20].

### Intestinal content collection and bacterial DNA extraction

Whole intestines of five zebrafish from each tank were aseptically dissected, and the intestinal content from each tank was pooled as one sample. Groups M and F contained five samples while Group E2 and BPA contained four samples. Total bacterial community DNA was isolated with an E.Z.N.A.™ Soil DNA Kit (Omega, USA). DNA yield was measured in a NanoDrop spectrophotometer. DNA quality was assessed by PCR amplification of the bacterial 16S rRNA gene.

### Illumina high-throughput sequencing of barcoded 16S rRNA genes

Bacterial DNA was used as the template for 16S rRNA gene V4-V5 region amplification [21]. The forward and reverse primers were 515F: 5ʹ-GTGCCAGCMGCCGCGGTAA-3ʹ and 907R: 5ʹ-CCGTCAATTCCTTTRAGTTT-3ʹ. Unique eight-base barcodes were added to each primer to distinguish PCR products. The PCR reaction mixture (25 μL) included 0.25 U of Platinum® Pfx DNA polymerase (Invitrogen), 2.5 μL of the corresponding 10×Pfx amplification buffer, 0.5 mM of MgSO_4_, 0.25 mM of deoxynucleoside triphosphate (dNTP) mixture, 6.25 pmol of each primer, and 20 ng of extracted DNA. The PCR program began with a 3 min denaturation step at 94°C, followed by 20 cycles of 1 min at 94°C (denaturation), a 1 min annealing step (65°C to 57°C with a 1°C reduction every two cycles followed by one cycle at 56°C and one cycle at 55°C), and a 1 min elongation step at 72°C; there was a final 6 min extension at 72°C [22]. Thirty nanograms of each purified PCR product were subjected to Illumina-based high-throughput sequencing (Majorbio Bio-Pharm Technology Co., Ltd., Shanghai, China). The sequences obtained in this paper are available in GeneBank with the accession number PRJNA304186.

### Bioinformatics and statistical analyzes

Raw fastq files were demultiplexed and quality-filtered using QIIME (version 1.17) [23]. Reads containing more than two mismatches to the primers or more than one mismatch to the barcode were discarded and reads with length <50 bp were removed. Reads (250 bp) were truncated at any site receiving an average quality score of <20 over a 50 bp sliding window.

Operational Taxonomic Units (OTUs) were clustered with a 97% similarity cutoff using UPARSE (version 7.1; http://drive5.com/uparse/) [24] and chimeric sequences were identified and removed using UCHIME [25]. The phylogenetic affiliation of each 16S rRNA gene sequence was analyzed by RDP Classifier (http://rdp.cme.msu.edu/) against the SILVA database using a confidence threshold of 70%.

Taxonomic richness and diversity estimators were determined for each library in Mothur. Rarefaction curves were created in Mothur to determine whether sequencing depth was sufficient to cover the expected number of OTUs at the level of 97% sequence similarity. ACE and Chao1 were used to reflect community richness [26,27]. Diversity was assessed using Shannon and Simpson indices [28,29]. All these indices were estimated based on OTU abundance matrices. The mean of the estimated parameters was used for comparisons among groups. Principal component analysis (PCA) was used to analyze all OTUs, affording information on microbial community differences among samples. Clustering analysis was based on the average OTU abundance in each group with Matlab R2013b. Network analysis was constructed by igraph package in R language to visualize the most abundant genus and to compare their abundance among groups. Statistical analysis was performed by one-way ANOVA followed by Tukey's post hoc multiple comparison test. P value < 0.05 was considered statistically significant.

## Results

### VTG expression level in livers of male fish exposed to E2 or BPA

**Fig 1:**
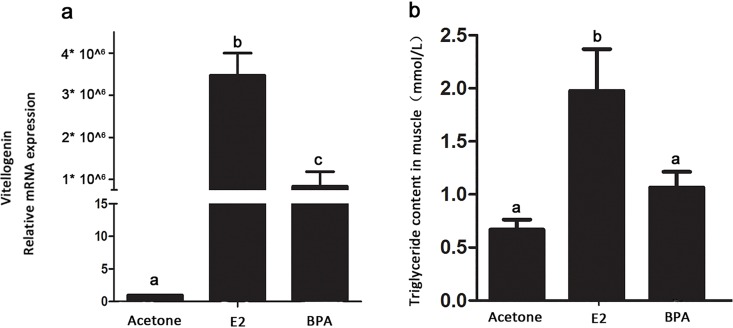
Two indices of the host evaluated the vitellogenin and triglyceride content in male zebrafish. a) The mRNA relative expression of VTG by E2 and BPA in male zebrafish liver. b) The triglyceride content in male zebrafish in muscle. Statistical analysis was carried out using one-way ANOVA. Different lower case letters represent significant differences among treatments (P< 0.05).

In order to avoid the influence of host estrogen, VTG expression level was detected on male zebrafish exposed to E2 or BPA at different concentrations for 5 weeks. As stated in the method, two concentration of BPA (2000μg/L) and E2 (2000ng/L) were used. Liver, as one of the most sexually dimorphic organs in terms of gene expression [30], and the main organ for production of VTG, was used for VTG expression level detection [31]. The results indicated that E2 and BPA significantly induced the expression of VTG in male zebrafish (F = 5.891, P = 0.0384) (Fig 1A), suggesting that E2 and BPA treatment caused female characters in male fish.

### TG content in muscles of male fish exposed to E2 or BPA

TG content of male zebrafish increased significantly in E2 treatment from 0.67±0.10 to 1.97±0.25 mmol/L (P = 0.0235). However, BPA treatment increased TG content in muscle from 0.67±0.10 to 1.06±0.15 mmol/L (P = 0.0889) (Fig 1B). All these results showed that BPA and E2 accelerated the TG accumulation in male fish.

### Microbial complexity of fish gut flora

A total of 236,006 quality reads were collected by Illumina high-throughput sequencing of barcoded bacterial 16S rRNA genes and used for subsequent data refinement. The rarefaction curves reached a plateau, suggesting good sampling depth (S3 Fig). Group BPA had the largest alpha-diversity indices (Ace, 100; Chao, 88; Shannon 1.63), followed by Group F, and then Group E2. Group M had the lowest alpha-diversity values (Ace, 82; Chao, 79; Shannon 1.26) (Table 1).

**Table 1:** Summary of intestinal microbiotic species richness estimators for sample groups.

Group	Reads	Gender	Ace	Chao	Shannon
**M**	12592	Male	82	79	1.26
**F**	11735	Female	88	85	1.47
**BPA**	11724	Male	100	88	1.63
**E2**	11201	Male	87	79	1.05

### Microbial community composition

**Fig 2:**
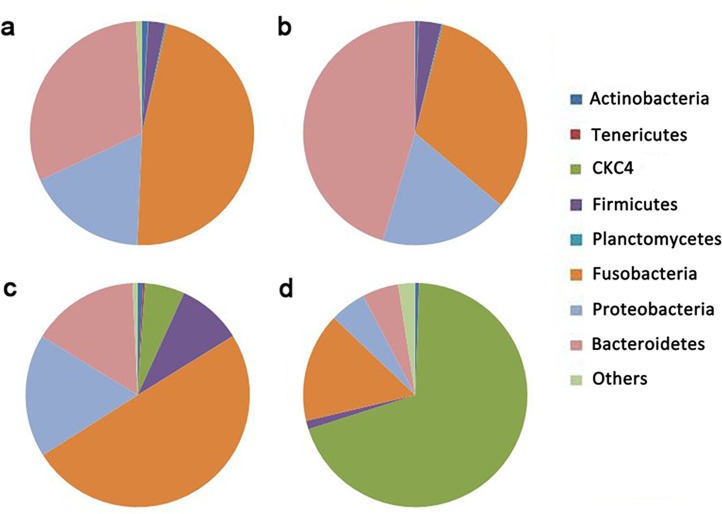
Intestinal microbiotic composition in zebrafish raised in different conditions. The pie charts represent the core microbiota of Group M (male) (a), Group F (female) (b), Group BPA (male treated with 2000 μg/L BPA) (c) and Group E2 (male treated with 2000 ng/L E2) (d).

Fig 2 illustrates bacterial community composition at the phylum level by pie charts. The predominant phyla in Groups M and F were Fusobacteria, Bacteroidetes and Proteobacteria. The abundances of these three phyla in Group M were 47.13%±8.45%, 31.00%±6.09% and 17.46%±0.46%, respectively, while in Group F, the abundance of these three phyla was32.19%±5.26%, 45.28%±0.01% and 18.56%±3.19%, respectively. The predominant phyla in Group BPA were Fusobacteria, Proteobacteria and Bacteroidetes, representing 49.83%±6.59%, 17.77%±2.41% and 15.50%±3.48% abundance, respectively. The predominated phyla in Group E2 were CKC4, Fusobacteria, Bacteroidetes and Proteobacteria, representing 69.53%±5.44%, 15.77%±3.58%, 5.25%±0.77% and 5.21%±1.16%, respectively. Most interestingly, abundance of CKC4 increased after the treatment with E2 and BPA from 0.05%±0.01% (Group M and Group F) to 5.74%±0.92% (Group BPA) and 69.53%±5.45% (Group E2) (P < 0.01). Other shared phyla in all groups included Firmicutes (1.22%±0.35%-9.38%±4.39%), Actinobacteria (0.44%±0.01%-0.84%±0.27%), Tenericutes (0.01%±0.01%-0.28%±0.01%) and Planctomycetes (0.00%±0.01%-0.19%±0.01%).

**Fig 3:**
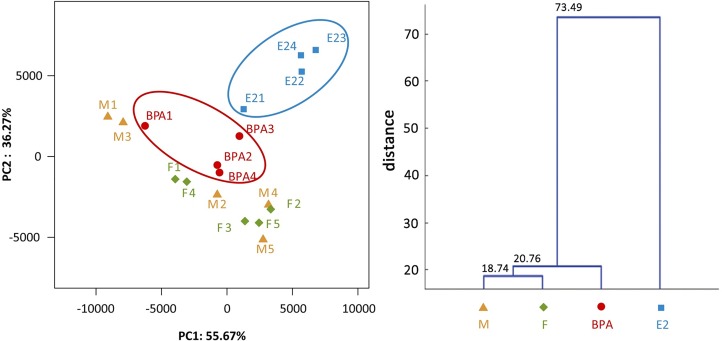
Overall structural comparison of intestinal microbiota in Groups M, F, BPA and E2. **a)** Principal component analysis scores plot. b) Clustering analysis of intestinal microbiota. The triangle, rhombus, roundness and square represented samples of Groups M, F, BPA and E2, respectively.

Principal component analysis was used to identify the intestinal microbial composition in four groups at the OTU level (Fig 3A). PC1 was 55.67% and PC2 was 36.27%. PCA plots showed that Groups F and M clustered together, consistent with our observation of a large shared microbiota between these two groups. Group E2 formed a distinct cluster and separated from the control group (Group M). Although Group BPA did not separate clearly from the control group, samples in Group BPA exhibited clear shifts from Groups M and F towards Group E2. These findings were also confirmed by the clustering analysis (Fig 3B).

**Fig 4:**
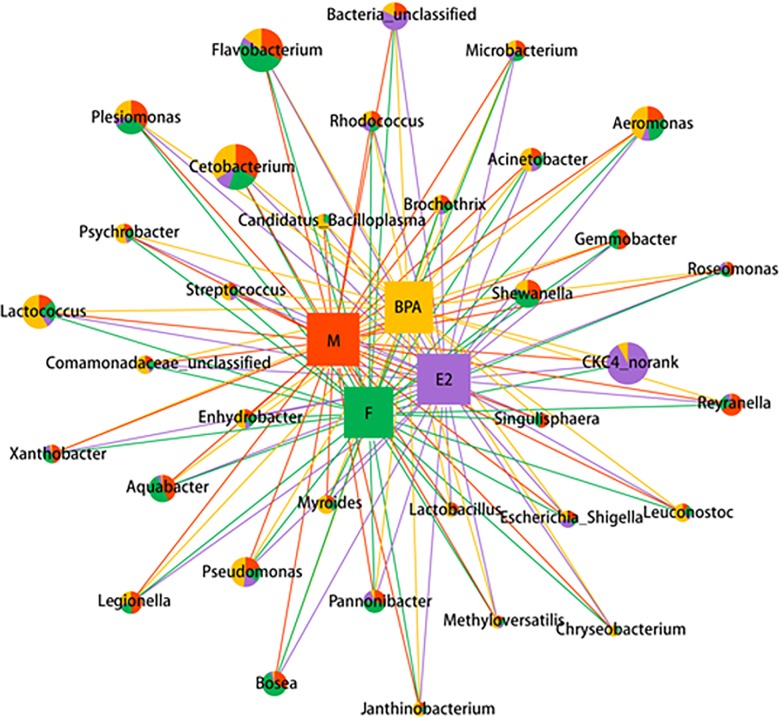
Network analysis visualizing the dominant genera in Groups M, F, BPA and E2. Node sizes correspond to the mean relative abundance of each genus. The proportions of genera shared by different groups are represented by the different colors in the pie charts.

Network analysis was used to summarize the proportion of genera in the intestinal microbiota of zebrafish subject to each treatment (Fig 4). Thirty-four genera with abundance>0.01%were selected for network analysis. Groups M and F shared a similar gut microbe composition. The dominant genera in all the groups were *Cetobacterium* and *Flavobacterium* whose abundance ranged from 26.87%±3.58% to 47.13%±8.45% and 4.62%±0.76% to 47.13%±2.64%, respectively. Four genera in Proteobacteria, namely *Acinetobacter*, *Aquabacter*, *Bosea* and *Xanthobacter*, decreased in Groups E2 and BPA compared with Groups M and F, while the proportion of CKC4 increased significantly in Groups BPA and E2 compared with Groups M and Group F.

## Discussion

Cardiovascular diseases [32], autoimmune diseases [10], human malaria [33] and many other diseases have been proven to be closely related to gender. Other evidences highlight the cross-reactivity among microbial flora, sex hormones and diseases in rodents and humans. For instance, in a tumor necrosis factor receptor 2 knock out (TNFR2^−/−^2D2) mouse model for central nervous system, demyelinating autoimmune disorders caused mostly female diseases development. Increased abundance of *Bacteroides sp*., *B*. *uniformis*, and *Parabacteroides sp*. in female mice and increased abundance of *Akkermansia muciniphila*, *Oscillospira sp*., *Bacteroides acidifaciens*, *Anaeroplasma sp*., and *Sutterella sp*. in male 2D2 mice suggested possible microbial influences on disease causation and protection [34]. It has been demonstrated that the gut community in male and female rats metabolized an oligo-fructose supplemented diet differently with an increase in the content of phylotypes of the phylum Bacteroidetes in female rats, while there were significantly higher levels of the pro-inflammatory cytokines IL-6 and CINC-1in male rats [35].

It was almost confirmed that the gut microbiota differs in men and women [36–38]. However, analysis of the intestinal microbiota in male and female zebrafish in the present study indicates that they share a similar bacterial composition (Fig 3). Consistent with the previous study [39], Fusobacteria and Proteobacteria are dominant members in both male and female zebrafish, suggesting that gender does not show significant influence on the intestinal microbiota in zebrafish. The possible reasons are:1) zebrafish are an undifferentiated gonochoristic species, with all individuals developing an immature ovarian tissue before the differentiation into mature ovaries or testes [40]; 2) zebrafish are exposed to the water environment from the embryo, so the microbial assembly is greatly impacted by water quality. Therefore, we assumed that water quality and the gonad development pathway played a more important role than host sex hormones in the assembly of gut microbiota in zebrafish.

In addition, we found that some studies in fish contained both genders also revealed the similar phenomenon, although no possible explanations had been given. Bacterial composition analysis in different intestine parts of Asian carp and indigenous American fish, with a sex ratio of half male and half female [41], showed no obvious gender-specific intestinal microbiota patterns. Another study conducted in zebrafish during different developmental stages also indicate that there is no significant effect of sex on the microbial community [42].

The powerful influence of water quality was not only restricted to gut microbiota but also applicable to skin microbiota in other aquatic animals including mammals. The data from a study on humpback whales (*Megaptera novaeangliae*) surface microbiota showed that there were non-significant distinctions between male and female whales [43], while it was found that men and women harbored distinct skin bacterial communities, even when controlling for hand hygiene [44].

After exposure of zebrafish to E2 and BPA, a transition to intersex happened in the treatment groups because of an increase in VTG content (Fig 1B). Surprisingly, both BPA and E2 altered the microbial constitution in a distinct manner from gender in PCA plots since female and male fish have similar microbiota to each other (Fig 3A). BPA and E2 treatment lead to a sharp increase in CKC4 abundance (Fig 2). It is a pity that the functional study of CKC4, a phylum in SLIVA database, is very limited, and our results suggested that CKC4 may be sensitive to estrogen and its analogue. The influence of E2 and BPA on the intestinal microbiota was similar, with higher estrogenic effect (E2: BPA = 10:1) resulting in greater distance from the “normal” microbiota in the PCA plot (Fig 3A). Furthermore, the triglyceride content in zebrafish muscle was increased by the BPA and E2 treatment (Fig 1A), which suggested that endocrine exposure and alteration of the intestinal microbiota may be related to changes in host lipid metabolism.

In conclusion, these findings showed that gut microbiota of male and female zebrafish were similar. Application of BPA and E2 resulted in bacterial dysbiosis, which may be related to changes in host lipid metabolism. These observations also suggest that intestinal microbiota may be one indicator for world-wide pollution by EDCs, although the exact mechanism remains unclear. Intestinal microbiota should be considered when we evaluate the influence of environment pollution or stress on the host health.

## Supporting Information

S1 Fig The mRNA relative expression of in male zebrafish liver with gradient concentration of EDCs. a) The treatment by 500ng/L E2 and 2000ng/L E2. b) The treatment by 200μg/L BPA and 2000μg/L BPA.
(DOCX)

S2 Fig The weight and the body length of sampled zebrafish. (DOCX)

S3 Fig Rarefaction analysis of Group M, Group F, Group BPA and Group E2. (DOCX)

## References

[1] Cani PD, Plovier H, Hul MV, Geurts L, Delzenne NM, et al (2016) Endocannabinoids—at the crossroads between the gut microbiota and host metabolism. Nat Rev Endocrinol 3:133–43. 10.1038/nrendo.2015.211 26678807

[2] Hevia A, Delgado S, Sanchez B, Margolles A (2015) Molecular Players Involved in the Interaction Between Beneficial Bacteria and the Immune System. Front Microbiol 6: 1285 10.3389/fmicb.2015.01285 26635753 PMC4649051

[3] Arentsen T, Raith H, Qian Y, Forssberg H, Diaz Heijtz R (2015) Host microbiota modulates development of social preference in mice. Microb Ecol Health Dis 26: 29719 10.3402/mehd.v26.29719 26679775 PMC4683992

[4] Engen PA, Green SJ, Voigt RM, Forsyth CB, Keshavarzian A (2015) The Gastrointestinal Microbiome: Alcohol Effects on the Composition of Intestinal Microbiota. Alcohol Res 37: 223–236. 26695747 10.35946/arcr.v37.2.07 PMC4590619

[5] Anahtar MN, Byrne EH, Doherty KE, Bowman BA, Yamamoto HS, et al (2015) Cervicovaginal bacteria are a major modulator of host inflammatory responses in the female genital tract. Immunity 42: 965–976. 10.1016/j.immuni.2015.04.019 25992865 PMC4461369

[6] Whary MT, Muthupalani S, Ge Z, Feng Y, Lofgren J, et al (2014) Helminth co-infection in Helicobacter pylori infected INS-GAS mice attenuates gastric premalignant lesions of epithelial dysplasia and glandular atrophy and preserves colonization resistance of the stomach to lower bowel microbiota. Microbes Infect 16: 345–355. 10.1016/j.micinf.2014.01.005 24513446 PMC4030519

[7] Boursi B, Mamtani R, Haynes K, Yang YX (2015) Recurrent antibiotic exposure may promote cancer formation—Another step in understanding the role of the human microbiota. Eur J Cancer 17:2655–64. 10.1016/j.ejca.2015.08.015 26338196 PMC4663115

[8] Adlercreutz H, Pulkkinen MO, Hamalainen EK, Korpela JT (1984) Studies on the role of intestinal bacteria in metabolism of synthetic and natural steroid hormones. J Steroid Biochem 20: 217–229. 10.1016/0022-4731(84)90208-5 6231418

[9] Flak MB, Neves JF, Blumberg RS (2013) Immunology. Welcome to the microgenderome. Science 339: 1044–1045. 10.1126/science.1236226 23449586 PMC4005781

[10] Markle JG, Frank DN, Mortin-Toth S, Robertson CE, Feazel LM, et al (2013) Sex differences in the gut microbiome drive hormone-dependent regulation of autoimmunity. Science 339: 1084–1088. 10.1126/science.1233521 23328391

[11] Gomez A, Luckey D, Taneja V (2015) The gut microbiome in autoimmunity: Sex matters. Clin Immunol 159: 154–162. 10.1016/j.clim.2015.04.016 25956531 PMC4560596

[12] Bhandari RK, Deem SL, Holliday DK, Jandegian CM, Kassotis CD, et al (2015) Effects of the environmental estrogenic contaminants bisphenol A and 17alpha-ethinyl estradiol on sexual development and adult behaviors in aquatic wildlife species. Gen Comp Endocrinol 214: 195–219. 10.1016/j.ygcen.2014.09.014 25277515

[13] Gilbert N (2012) Drug-pollution law all washed up. Nature 491: 503–504. 10.1038/491503a 23172189

[14] Bjerregaard LB, Lindholst C, Korsgaard B, Bjerregaard P (2008) Sex hormone concentrations and gonad histology in brown trout (Salmo trutta) exposed to 17beta-estradiol and bisphenol A. Ecotoxicology 17: 252–263. 10.1007/s10646-008-0192-2 18320304

[15] Valencia A, Rojo-Bartolome I, Bizarro C, Cancio I, Ortiz-Zarragoitia M (2016) Alteration in molecular markers of oocyte development and intersex condition in mullets impacted by wastewater treatment plant effluents. Gen Comp Endocrinol. 10.1016/j.ygcen.2016.06.017 27296671

[16] Staples CA, Dorn PB, Klecka GM, O'Block ST, Harris LR (1998) A review of the environmental fate, effects, and exposures of bisphenol A. Chemosphere 36: 2149–2173. 10.1016/s0045-6535(97)10133-3 9566294

[17] Witorsch RJ (2002) Endocrine disruptors: can biological effects and environmental risks be predicted? Regul Toxicol Pharmacol 36: 118–130. 10.1006/rtph.2002.1564 12383724

[18] Inagaki T, Smith N, Lee EK, Ramakrishnan S (2016) Low dose exposure to Bisphenol A alters development of gonadotropin-releasing hormone 3 neurons and larval locomotor behavior in Japanese Medaka. Neurotoxicology 52: 188–197. 10.1016/j.neuro.2015.12.003 26687398

[19] Saili KS, Corvi MM, Weber DN, Patel AU, Das SR, et al (2012) Neurodevelopmental low-dose bisphenol A exposure leads to early life-stage hyperactivity and learning deficits in adult zebrafish. Toxicology 291: 83–92. 10.1016/j.tox.2011.11.001 22108044 PMC3245816

[20] Trinder P (1969) Determination of glucose in blood using glucose oxidase with an alternative oxygen acceptor. Ann Clin Biochem 6: 24–33. 10.1177/000456326900600108

[21] Sun DL, Jiang X, Wu QL, Zhou NY (2013) Intragenomic heterogeneity of 16S rRNA genes causes overestimation of prokaryotic diversity. Appl Environ Microbiol 79: 5962–5969. 10.1128/AEM.01282-13 23872556 PMC3811346

[22] Gunimaladevi I, Savan R, Sakai M (2006) Identification, cloning and characterization of interleukin-17 and its family from zebrafish. Fish Shellfish Immunol 21: 393–403. 10.1016/j.fsi.2006.01.004 16677828

[23] Caporaso JG, Kuczynski J, Stombaugh J, Bittinger K, Bushman FD, et al (2010) QIIME allows analysis of high-throughput community sequencing data. Nat Methods 7: 335–336. 10.1038/nmeth.f.303 20383131 PMC3156573

[24] Edgar RC (2013) UPARSE: highly accurate OTU sequences from microbial amplicon reads. Nat Methods 10: 996–998. 10.1038/nmeth.2604 23955772

[25] Edgar RC, Haas BJ, Clemente JC, Quince C, Knight R (2011) UCHIME improves sensitivity and speed of chimera detection. Bioinformatics 27: 2194–2200. 10.1093/bioinformatics/btr381 21700674 PMC3150044

[26] Chao A (1984) Nonparametric estimation of the number of classes in a population. Scand J Stat 11: 265–270.

[27] Chao A, Lee SM (1992) Estimating the bumber of classes cia sample coverage. J Am Stat Assoc: 210–217. 10.1080/01621459.1992.10475194

[28] Shannon CE (1948) A mathematical theory of communication. Bell Syst Tach J 27: 379–423.

[29] Simpson EH (1949) Measurement of diversity. Nature 163: 688.

[30] Yang X, Schadt EE, Wang S, Wang H, Arnold AP, et al (2006) Tissue-specific expression and regulation of sexually dimorphic genes in mice. Genome Res 16: 995–1004. 10.1101/gr.5217506 16825664 PMC1524872

[31] Zheng W, Xu H, Lam SH, Luo H, Karuturi RK, et al (2013) Transcriptomic analyses of sexual dimorphism of the zebrafish liver and the effect of sex hormones. PLoS One 8: e53562 10.1371/journal.pone.0053562 23349717 PMC3547925

[32] Leifheit-Limson EC, D'Onofrio G, Daneshvar M, Geda M, Bueno H, et al (2015) Sex Differences in Cardiac Risk Factors, Perceived Risk, and Health Care Provider Discussion of Risk and Risk Modification Among Young Patients With Acute Myocardial Infarction: The VIRGO Study. J Am Coll Cardiol 66: 1949–1957. 10.1016/j.jacc.2015.08.859 26515996 PMC4628727

[33] Dkhil MA, Al-Shaebi EM, Lubbad MY, Al-Quraishy S (2015) Impact of sex differences in brain response to infection with Plasmodium berghei. Parasitol Res. 10.1007/s00436-015-4803-6 26499384

[34] Miller PG, Bonn MB, Franklin CL, Ericsson AC, McKarns SC (2015) TNFR2 Deficiency Acts in Concert with Gut Microbiota To Precipitate Spontaneous Sex-Biased Central Nervous System Demyelinating Autoimmune Disease. J Immunol 10:4668–84. 10.4049/jimmunol.1501664 26475926 PMC8449043

[35] Shastri P, McCarville J, Kalmokoff M, Brooks SP, Green-Johnson JM (2015) Sex differences in gut fermentation and immune parameters in rats fed an oligofructose-supplemented diet. Biol Sex Differ 6: 13 10.1186/s13293-015-0031-0 26251695 PMC4527341

[36] Cong X, Xu W, Janton S, Henderson WA, Matson A, et al (2016) Gut Microbiome Developmental Patterns in Early Life of Preterm Infants: Impacts of Feeding and Gender. PLoS One 11: e0152751 10.1371/journal.pone.0152751 27111847 PMC4844123

[37] Haro C, Rangel-Zuniga OA, Alcala-Diaz JF, Gomez-Delgado F, Perez-Martinez P, et al (2016) Intestinal Microbiota Is Influenced by Gender and Body Mass Index. PLoS One 11: e0154090 10.1371/journal.pone.0154090 27228093 PMC4881937

[38] Singh P, Manning SD (2016) Impact of age and sex on the composition and abundance of the intestinal microbiota in individuals with and without enteric infections. Ann Epidemiol 26: 380–385. 10.1016/j.annepidem.2016.03.007 27118420 PMC9494358

[39] Roeselers G, Mittge EK, Stephens WZ, Parichy DM, Cavanaugh CM, et al (2011) Evidence for a core gut microbiota in the zebrafish. ISME J 5: 1595–1608. 10.1038/ismej.2011.38 21472014 PMC3176511

[40] Luzio A, Monteiro SM, Garcia-Santos S, Rocha E, Fontainhas-Fernandes AA, et al (2015) Zebrafish sex differentiation and gonad development after exposure to 17alpha-ethinylestradiol, fadrozole and their binary mixture: A stereological study. Aquat Toxicol 166: 83–95. 10.1016/j.aquatox.2015.07.015 26240953

[41] Ye L, Amberg J, Chapman D, Gaikowski M, Liu WT (2014) Fish gut microbiota analysis differentiates physiology and behavior of invasive Asian carp and indigenous American fish. ISME J 8: 541–551. 10.1038/ismej.2013.181 24132079 PMC3930320

[42] Stephens WZ, Burns AR, Stagaman K, Wong S, Rawls JF, et al (2015) The composition of the zebrafish intestinal microbial community varies across development. ISME J 3:644–54. 10.1038/ismej.2015.140 26339860 PMC4817687

[43] Apprill A, Mooney TA, Lyman E, Stimpert AK, Rappe MS (2011) Humpback whales harbour a combination of specific and variable skin bacteria. Environ Microbiol Rep 3: 223–232. 10.1111/j.1758-2229.2010.00213.x 23761254

[44] Fierer N, Hamady M, Lauber CL, Knight R (2008) The influence of sex, handedness, and washing on the diversity of hand surface bacteria. Proc Natl Acad Sci U S A 105: 17994–17999. 10.1073/pnas.0807920105 19004758 PMC2584711

