# Supplementary material for: Influence of Endogenous and Exogenous Estrogenic Endocrine on Intestinal Microbiota in Zebrafish
Source: PLoS One. 2016 Oct 4;11(10):e0163895. doi: 10.1371/journal.pone.0163895 (PMC5049800; doi:10.1371/journal.pone.0163895)
Supplement: S1 Fig — a) The treatment by 500ng/L E2 and 2000ng/L E2. b) The treatment by 200μg/L BPA and 2000μg/L BPA. (DOCX) [file pone.0163895.s001.docx]

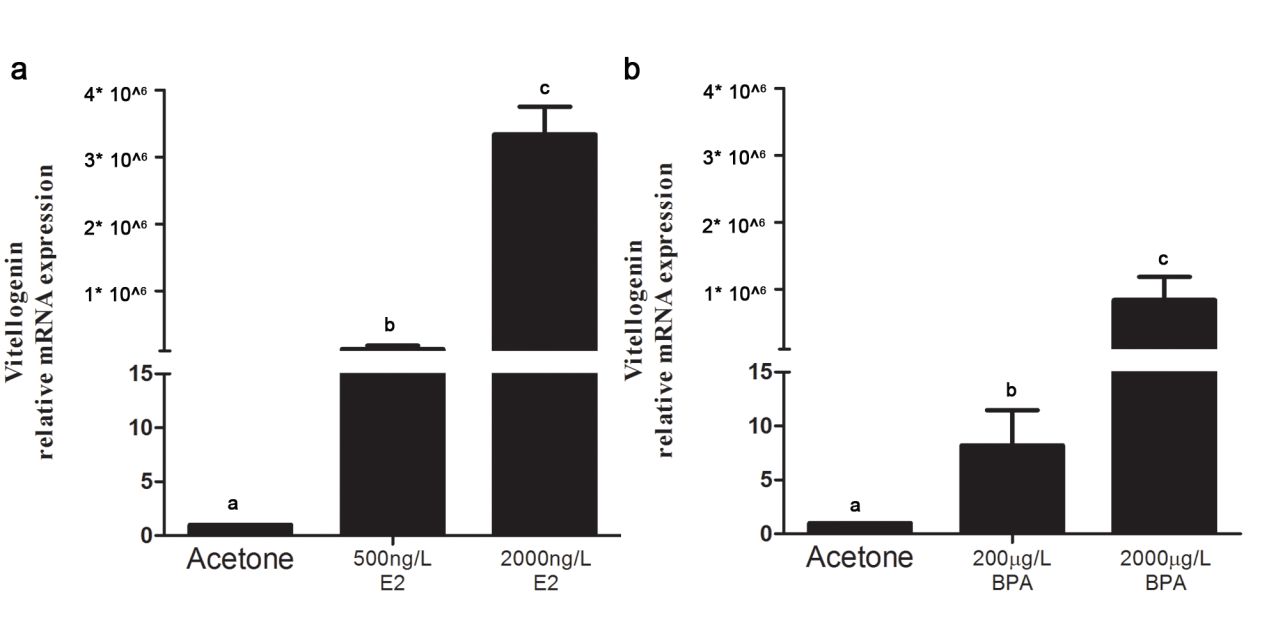


**S1 Fig.** The mRNA relative expression of in male zebrafish liver with gradient concentration of EDCs. a) The treatment by 500ng/L E2 and 2000ng/L E2. b) The treatment by 200µg/L BPA and 2000µg/L BPA.
