# Supplementary figures and images for: Influence of Endogenous and Exogenous Estrogenic Endocrine on Intestinal Microbiota in Zebrafish
Source: PLoS One. 2016 Oct 4;11(10):e0163895. doi: 10.1371/journal.pone.0163895 (PMC5049800; doi:10.1371/journal.pone.0163895)

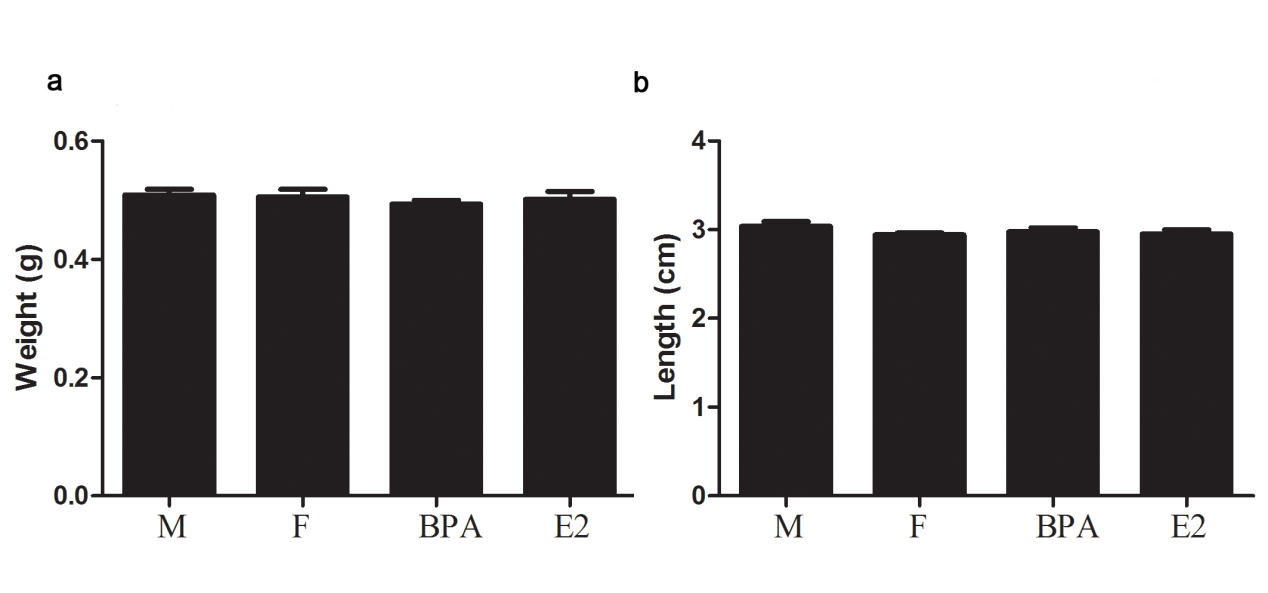


**S2 Fig.** The weight and the body length of sampled zebrafish.

Supplement: S2 Fig — (DOCX) [file pone.0163895.s002.docx]

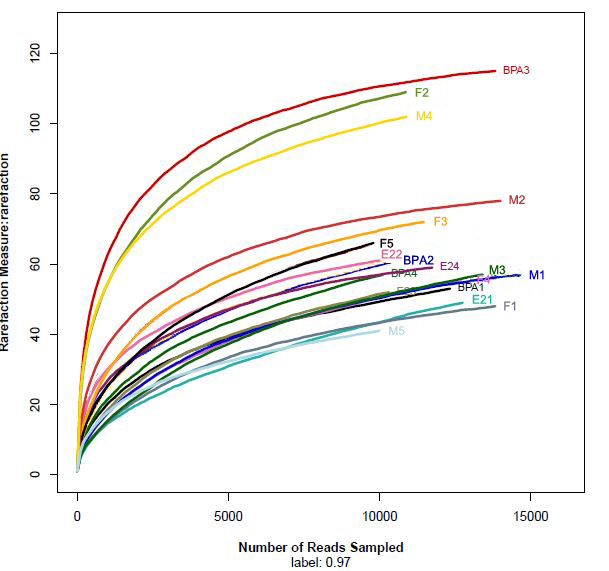


**S3** **Fig.** Rarefaction analysis of Group M (n=5), Group F (n=5), Group BPA (n=4) and Group E2 (n=4).

Supplement: S3 Fig — (DOCX) [file pone.0163895.s003.docx]
